# The impact of nutritional support therapy combined with conventional treatment models on short-term symptom improvement and complications in stroke patients: a systematic review and meta-analysis

**DOI:** 10.3389/fnut.2025.1642161

**Published:** 2025-11-11

**Authors:** Meng Zhang, Meng Li, Ying Ding, Yi Zhang, Li Zhang, Xiapei Peng

**Affiliations:** Department of Neurology, The Central Hospital of Wuhan, Tongji Medical College, Huazhong University of Science and Technology, Wuhan, China

**Keywords:** nutritional support therapy, conventional treatment, stroke, long-term prognosis, meta-analysis

## Abstract

**Objective:**

To methodically assess the effectiveness of nutritional support therapy combined with conventional treatment on short-term symptom improvement, nutritional and immune recovery, and complication rates in stroke patients.

**Methods:**

A thorough literature search was carried out utilizing PubMed, EMBASE, ScienceDirect, the Cochrane Library, and major Chinese databases (CNKI, VIP, Wanfang, and CBM) from inception to the present. Randomized controlled trials (RCTs) evaluating the impact of nutritional support in stroke patients were included. Two reviewers independently extracted the data, and the Cochrane Handbook 5.3 was used to determine the risk of bias. RevMan 5.3 was used to conduct the meta-analysis.

**Results:**

Following PRISMA guidelines, 1,693 records were retrieved and screened, resulting in the inclusion of 8 randomized controlled trials with a total of 727 individuals. Meta-analysis revealed that nutritional support significantly improved Glasgow Coma Scale (GCS) scores, serum markers of nutritional status (Hb, TLC), and immune parameters (IgA, IgG, IgM). Pro-inflammatory cytokines (IL-2, IL-6, TNF-*α*) were significantly reduced. Moreover, the incidence of infectious complications was lower in the intervention group. However, heterogeneity among studies was high in several analyses, warranting cautious interpretation.

**Conclusion:**

Nutritional support combined with conventional therapy improves nutritional and immune recovery and reduces infection risk in stroke patients. However, given the high heterogeneity and methodological limitations of included trials, the certainty of evidence remains low to very low, and these results should be interpreted cautiously.

## 1 Introduction

Stroke is a common cerebrovascular disorder frequently encountered in clinical practice. With the global trend of population aging, the incidence of stroke continues to rise annually. Stroke can lead to a range of motor impairments due to damage to the central nervous system, with dysphagia being one of the most prevalent complications (1). According to published literature (2, 3), approximately 22 to 65% of stroke patients develop dysphagia. Affected individuals may experience impaired swallowing or an increased risk of regurgitation and aspiration, which compromises nutritional intake and absorption and significantly elevates the risk of aspiration pneumonia. Moreover, stroke is often associated with cerebral edema, elevated intracranial pressure, neurological dysfunction, and reduced gastrointestinal motility—all of which further impair nutritional intake, weaken immune function, delay neurological recovery, increase mortality risk, and prolong hospitalization (4).

Nutritional support therapy, including enteral nutrition, parenteral nutrition and combined application, has a significant part in improving the nutritional status of stroke individuals, reducing complications and promoting functional recovery (5, 6). Among them, early enteral nutrition (EN) shortens the establishment time of sitting balance and standing balance by maintaining intestinal barrier function, regulating immune response, and reducing the risk of infection. Combined enteral and parenteral nutritional support (EN + PN) can rapidly correct hypoproteinemia, improve the levels of total plasma protein, albumin and hemoglobin, and reduce the incidence of complications such as pneumonia and pressure ulcers. The patients who received early nutritional intervention (within 72 h of onset) had significantly better modified Barthel index scores and the degree of improvement in neurological deficits than those in the delayed intervention group (IG) (7). However, existing studies show heterogeneity in terms of the types of nutritional support, the timing of intervention and evaluation indicators. For instance, some studies have strongly adjusted the short-term metabolic advantages of protein-based enteral nutrition agents (8), while others recommend sequential enteral nutrition to reduce the risk of infection (9). Based on the above background, this article seeks to systematically evaluate the effect of nutritional support therapy applied to stroke patients through evidence-based methods, aiming to provide a reference basis for clinicians to formulate more scientific and reasonable diagnosis and treatment measures.

The objective of this systematic review and meta-analysis was to evaluate the short-term effects of nutritional support therapy combined with conventional treatment on neurological recovery, nutritional and immune function, and infection-related complications in patients with stroke.

## 2 Methods

### 2.1 The sources and procedures used to get literary materials

A comprehensive literature search was conducted using electronic databases, including PubMed, EMBASE, ScienceDirect, the Cochrane Library, the China National Knowledge Infrastructure (CNKI), the Chinese Biomedical Literature Database (CBM), the Wanfang Database, and the VIP Full-Text Database. In addition, manual searches were performed to identify relevant studies from Chinese and international journals, conference proceedings, dissertations, and other academic sources. Reference lists of retrieved articles were also reviewed to identify additional relevant publications.

The search focused on studies assessing the effects of nutritional support therapy on symptom improvement and long-term prognosis in patients with stroke. The following Boolean search strategy was employed to ensure comprehensive coverage:

(“nutritional support therapy” OR “nutritional intervention” OR “enteral nutrition” OR “parenteral nutrition” OR “clinical nutrition support”) AND (“routine treatment” OR “conventional therapy”) AND (“stroke” OR “cerebral infarction” OR “cerebral hemorrhage”) AND (“prognosis” OR “recovery” OR “rehabilitation outcome”).

Both Medical Subject Headings (MeSH) and free-text terms were utilized. Search terms and operators were adapted according to the specific syntax requirements of each database. The search was restricted to publications dated from January 2010 to the present.

### 2.2 Criteria for literature inclusion and exclusion

#### 2.2.1 Inclusion criteria of literature

To ensure a comprehensive and structured selection process, inclusion criteria were defined according to the PICOS framework:

Population (P): Adult patients (≥18 years) clinically diagnosed with stroke (ischemic or hemorrhagic) according to established diagnostic criteria. Studies were excluded if participants required palliative care, had acute coronary syndrome, transient ischemic attack, subarachnoid hemorrhage, progressive neurological diseases, heart failure, or respiratory failure, or if they had pre-existing disability before stroke onset.

Intervention (I): Nutritional support therapy, including enteral nutrition, nasogastric nutritional management, parenteral nutrition, or combined enteral–parenteral approaches. Studies describing early nutritional support (initiated within 72 h) or immune-enhanced formulations were also eligible.

Comparator (C): Routine or conventional treatment without structured nutritional support, or family-based nutritional management consistent with standard hospital care.

Outcomes (O): Primary outcomes: Nutritional status indicators {[serum prealbumin (PA), albumin (Alb), total lymphocyte count (TLC), and hemoglobin (Hb)]}|.

Secondary outcomes: Neurological function {[National Institutes of Health Stroke Scale (NIHSS)] score}, level of consciousness {[Glasgow Coma Scale (GCS)] score}, immune function markers (IgA, IgG, IgM), inflammatory cytokines {[tumor necrosis factor-alpha (TNF-*α*), interleukin-2 (IL-2), and interleukin-6 (IL-6)]}, and incidence of infectious complications.

**Figure 1:**
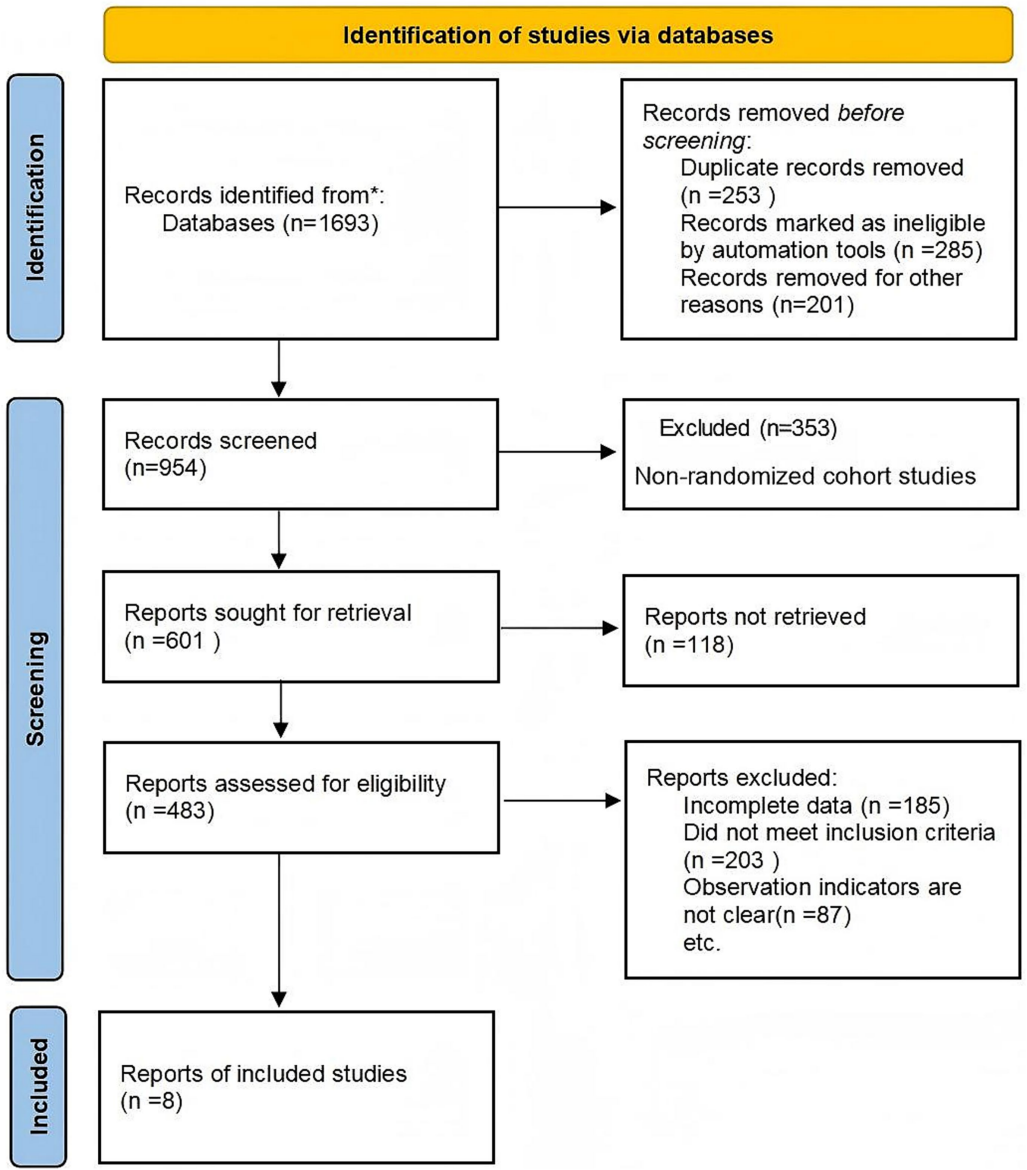
Flowchart of literature screening.

Study design (S): Randomized controlled trials (RCTs) were included in the final analysis to ensure methodological rigor. Although cohort studies were initially considered during the search stage for comprehensiveness, only RCTs meeting the Cochrane Handbook 5.3 criteria were retained after quality assessment. Although cohort studies were initially considered to capture a broad evidence base, they were excluded after quality appraisal because they lacked randomization or comparable control groups, as reflected in the PRISMA flowchart (Figure 1).

All included studies reported baseline patient characteristics and intervention details. However, the reporting of blinding and attrition varied among studies, which was considered in the risk of bias assessment.

Efficacy Endpoints: Neurological function: Assessed using the NIHSS, that falls between 0 and 42 points. Higher scores show more serious neurological impairments, with 0–1 indicating normal or mild deficits and ≥21 indicating severe impairment (10). Level of consciousness: Evaluated using the GCS, with a total score of 15. Lower scores indicate deeper levels of coma (11). Nutritional status: Assessed via PA, Alb, TLC, and Hb. Immune function: Measured by serum levels of immunoglobulins (IgA, IgM, IgG). Inflammatory markers: Including TNF-*α*, IL-2, and IL-6.

#### 2.2.2 Literature exclusion criteria

(1) Research not involving randomized controlled trials.
(2) Studies with incomplete or non-usable data.
(3) Duplicate publications (only the most recent version was retained).
(4) Studies lacking clearly defined outcomes.
(5) Review articles, meta-analyses, or theoretical literature.
(6) Case reports or clinical case series.

### 2.3 Quality evaluation and data extraction

#### 2.3.1 Risk of bias assessment

The Cochrane Collaboration’s “Risk of Bias” assessment technique, described in the Cochrane Handbook for Systematic Reviews of Interventions, version 5.3, was used to assess the risk of bias in the included studies.

#### 2.3.2 Literature screening and data extraction

The literature was separately examined, pertinent data was retrieved, and the quality of the study was evaluated by two reviewers. Any disagreements were settled by discussion or, if required, by a third reviewer. NoteExpress and Microsoft Excel were used for reference management and data extraction. Attempts were made to get in touch with the original writers for clarification or further information in situations where crucial material was unclear or missing. The information that was retrieved contained: (1) Basic study information: first author, year of publication, and sample size; (2) Intervention details: Nutritional support therapy (e.g., enteral nutrition, nasogastric feeding) and conventional treatment (standard nutritional care). When reported, data on the enteral-to-parenteral nutrition (EN/PN) ratio, nutrient composition (such as protein- or peptide-based formulations, lipid emulsions, immunonutrient-enriched formulas), and timing and duration of initiation were also extracted. However, reporting across studies was inconsistent, with several trials lacking detailed specification of formula type, caloric density, or supplementation content; (3) Outcome measures: Neurological function (NIHSS score), level of consciousness (GCS score), nutritional status markers (prealbumin, albumin, hemoglobin, total lymphocyte count), immune function (IgA, IgM, IgG), inflammatory biomarkers (IL-2, IL-6, TNF-*α*), and incidence of infectious complications.

### 2.4 Statistical processing

The Cochrane Collaboration developed the RevMan 5.4 program for meta-analysis. Counting data adopted the Odds Ratio (OR) as the effect indicator. RevMan 5.4 was used to analyze data, including the incidence of infectious complications, serum inflammatory factor levels, nutritional status indicators, immunological function indicators, NIHSS score, and GCS score. For dichotomous outcomes (incidence of infectious complications), effect sizes were calculated as odds ratios (ORs) with 95% confidence intervals (CIs). For continuous outcomes (NIHSS, GCS, PA, Alb, Hb, TLC, IgA, IgG, IgM, IL-2, IL-6, TNF-*α*), effect sizes were calculated as mean differences (MDs) with 95% CIs. Fixed- or random-effects models were selected according to between-study heterogeneity (χ^2^ test and I^2^ statistic). The included studies were deemed homogenous if *p* > 0.1 and I^2^ < 50%, and the adjusted influence models could be gathered for meta-analysis. When evaluating the homogeneity of the included studies, the random effects model was chosen if *p* < 0.1 and I^2^ > 50% and a combined effect was required. When P is less than 0.1 and the source of heterogeneity cannot be identified, descriptive analysis is used instead of meta-analysis. To further examine the publication bias of the included literature, an inverted funnel plot was created. Since the number of literatures included in this study was less than 10, funnel plot drawing was not conducted.

Given the anticipated clinical and methodological heterogeneity across studies, exploratory subgroup and sensitivity analyses were prespecified. Subgroups of interest comprised: (1) route and composition of nutritional support (enteral nutrition alone vs. nasogastric nutrition management vs. combined or immunonutrient-enriched formulations, where reported); (2) timing and duration of the intervention (early enteral nutrition initiated during the acute phase vs. later initiation; short-term courses ≤2 weeks vs. longer durations); (3) stroke subtype and severity (hemorrhagic vs. ischemic or mixed types; severe vs. non-severe cases, where reported); and (4) control regimen (conventional care vs. family-administered nutrition management).

Sensitivity analyses included leave-one-out analyses, exclusion of small-sample studies, and restriction to studies with comparable laboratory measurement protocols. In cases of insufficient data or inconsistent definitions across studies, subgroup pooling was not performed, and relevant findings were summarized narratively.

## 3 Results and analysis

### 3.1 The outcomes of the literature search and the fundamental state of the included literature

The literature review followed Preferred Reporting Items for Systematic Reviews and Meta-Analyses (PRISMA) guidelines. A total of 1,693 records were retrieved; after screening and full-text assessment, non-randomized cohort studies were excluded due to methodological limitations and absence of comparable control groups. Ultimately, eight RCTs involving 727 participants were included in the meta-analysis (Figure 1).

Table 1 presents the basic characteristics of the eight included RCTs, including study design, diagnostic methods, conventional and nutritional interventions, demographic characteristics, and primary results. Most studies implemented early enteral nutrition as the main intervention, while control groups received conventional treatment or family-based nutritional management. Despite similarities in population and intervention timing, the composition and dosage of nutritional support varied considerably, contributing to potential clinical heterogeneity.

**Table 1:** Basic characteristics of the included randomized controlled trials.

References	Study design	Diagnostic method	Sample size (CG/IG)	Conventional treatment	Nutritional support (type, components, duration)	Demographics (mean age, % male)	Main statistical results	Conclusion
Zhuo Yurong (17)	RCT	CT/MRI-confirmed hemorrhagic stroke with dysphagia	44/44	Routine medical care + rehabilitation	Enteral nutrition with individualized energy targets, protein 1.2 g/kg/day, duration 14 days	63.2 ± 8.7; 54.5% male	Alb↑, PA↑, IgG↑, TNF-α↓ (*P* < 0.05)	Nutritional support improved nutritional and immune status, reduced complications
Sun Maling (15)	RCT	Clinical + CT confirmation	36/36	Family-based nutrition guidance	Early EN (nasogastric feeding, polymeric formula, 20–25 kcal/kg/day)	65.4 ± 7.2; 58.3% male	Hb↑, IgA↑, TNF-α↓ (*P* < 0.05)	Early EN improved nitrogen balance and immune recovery
Qingzhi (22)	RCT	CT/MRI confirmed severe stroke	42/42	Routine medical + dehydration + anti-infection	Early EN, 1500 kcal/day, supplemented with ω-3 fatty acids	64.1 ± 9.0; 50% male	IgM↑, IL-6↓ (*P* < 0.05)	EN improved immune function and reduced inflammation
Xi Junnan and Huiyuan (23)	RCT	CT/MRI-confirmed severe stroke	41/41	Routine therapy + rehabilitation	EN (standard formula, gradual progression to full feeding, 21 days)	62.8 ± 8.4; 53.7% male	IgG↑, IL-2↑ (*P* < 0.05)	EN modulated immune and inflammatory response
Rao Liumei (12)	RCT	CT-confirmed stroke	46/46	Routine treatment + basic care	Early EN (nasogastric, 1.5 g protein/kg/day)	63.7 ± 9.3; 51.2% male	Alb↑, IgA↑, infection rate↓ (*P* < 0.05)	Early EN improved nutrition and reduced infection
Youai (16)	RCT	Clinical + imaging diagnosis	34/35	Routine treatment	Early EN (polymeric formula, 10–14 days)	66.1 ± 7.8; 47% male	Alb↑, infection↓ (*P* < 0.05)	Early EN accelerated recovery
Li Li (13)	RCT	CT-confirmed severe stroke	44/50	Routine medical therapy + dehydration	EN (immune-enhanced formula, ω-3 fatty acids, 14 days)	64.5 ± 8.1; 55% male	IgG↑, IL-6↓ (*P* < 0.05)	Immune EN improved inflammation and nutritional indicators
Zheng et al. (14)	RCT	CT/MRI-confirmed acute stroke	71/75	Family nutrition management	Nasogastric feeding (1,800 kcal/day, 10 days)	65.3 ± 9.1; 56.8% male	Infection rate↓ (*P* < 0.05)	Early NG feeding improved short-term prognosis

EN, enteral nutrition; PN, parenteral nutrition; Alb, albumin; PA, prealbumin; Ig, immunoglobulin; TNF-α, IL, inflammatory cytokines.

### 3.2 Assessment of the methodological quality of the literature

**Figure 2:**
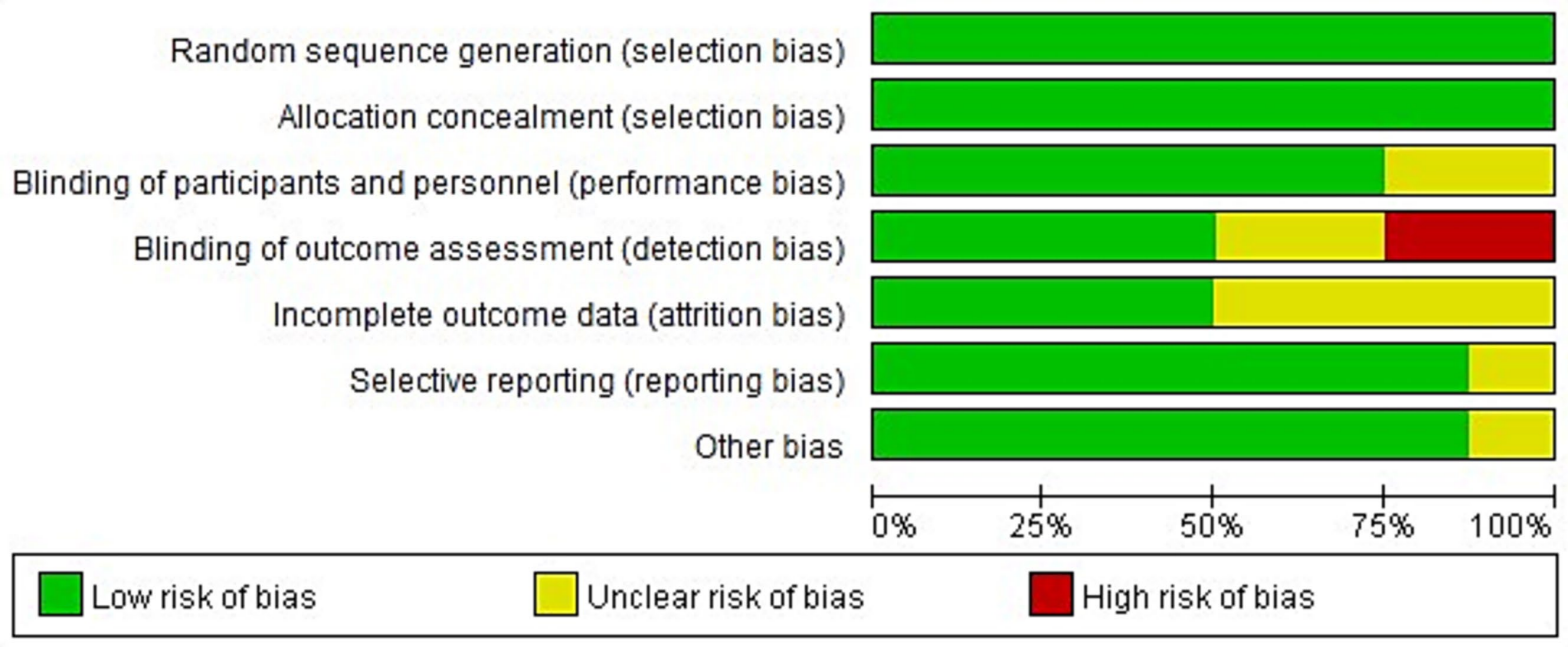
Risk bias graph.

**Figure 3:**
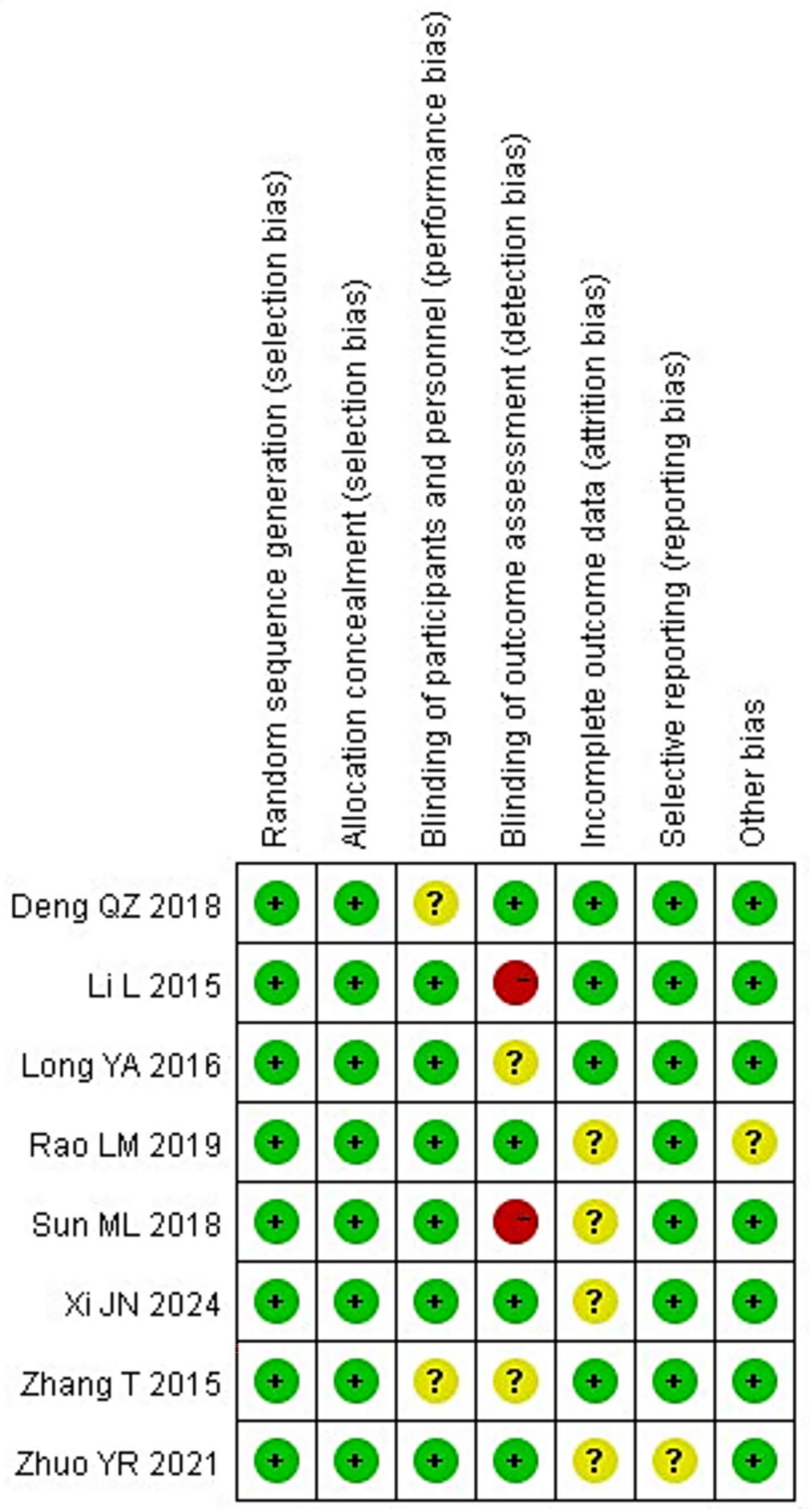
Summary chart of risk bias.

All eight of the RCTs that were included of this meta-analysis reported baseline patient information and gave thorough explanations of the intervention procedures and outcome measures. However, none of the studies explicitly reported the methods or extent of blinding, nor did they adequately describe the number of individuals lost to follow-up or the reasons for withdrawal. Figures 2, 3 show the risk of bias assessment for the included studies.

Quantitatively, six of the eight studies (75%) were judged as having a low risk of bias for random sequence generation and allocation concealment, while all studies were rated as having unclear or high risk for blinding of participants and outcome assessment. Specifically, 2 studies (25%) showed a high risk of performance bias due to unblinded interventions, and 3 studies (37.5%) had unclear detection bias. Incomplete outcome data were reported in only 3 studies (37.5%), and selective reporting was unclear in 2 (25%).

The overall methodological quality of the included RCTs was suboptimal. None of the trials explicitly reported the use or extent of blinding, and most failed to describe participant attrition or reasons for withdrawal. These deficiencies raise a high risk of performance and detection bias, especially for outcomes that rely on subjective clinical judgment or additional attention (e.g., GCS scores and infection monitoring). Therefore, while pooled analyses suggest beneficial effects, the certainty of evidence for all main outcomes should be considered low to very low.

### 3.3 Meta-analysis results

#### 3.3.1 Neural function

**Figure 4:**
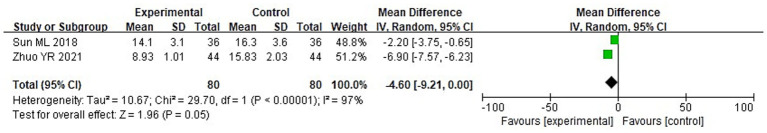
Forest analysis diagrams comparing the NIHSS scores of the two groupings.

A total of 8 studies were included in this research, involving a total of 727 samples. Among them, 2 literatures reported the NIHSS scores of the two groups after treatment. It can be known from the findings of the heterogeneity test that: Chi^2^ = 29.70, df = 1, *p* < 0.00001, I^2^ = 97%, indicating significant heterogeneity among the included research data. Analysis using the random effects model shows (Figure 4) that there was no discernible variation in the NIHSS scores involving the two groupings of individuals (MD = −4.60, 95%CI: −9.21 to 0.00, *p* = 0.05).

#### 3.3.2 Degree of coma

**Figure 5:**
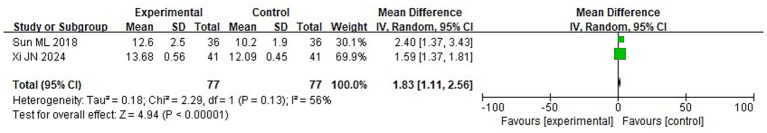
Forest analysis chart comparing the GCS scores of the two groups.

Among 8 studies, 2 studies reported the GCS scores of the two groups after treatment. It can be known from the findings of the heterogeneity test that: Chi^2^ = 2.29, df = 1, *p* = 0.13, I^2^ = 56%, indicating heterogeneity among the included research data. It can be known from the random effects model analysis (Figure 5) that the GCS score of the IG after treatment was higher than that of the control group (CG) (MD = 1.83, 95%CI: 1.11–2.56, *p* < 0.0001).

#### 3.3.3 Nutritional status

**Figure 6:**
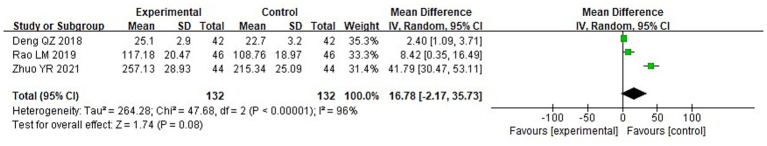
Forest analysis diagram comparing the levels of prealbumin (PA) in the two groupings.

**Figure 7:**
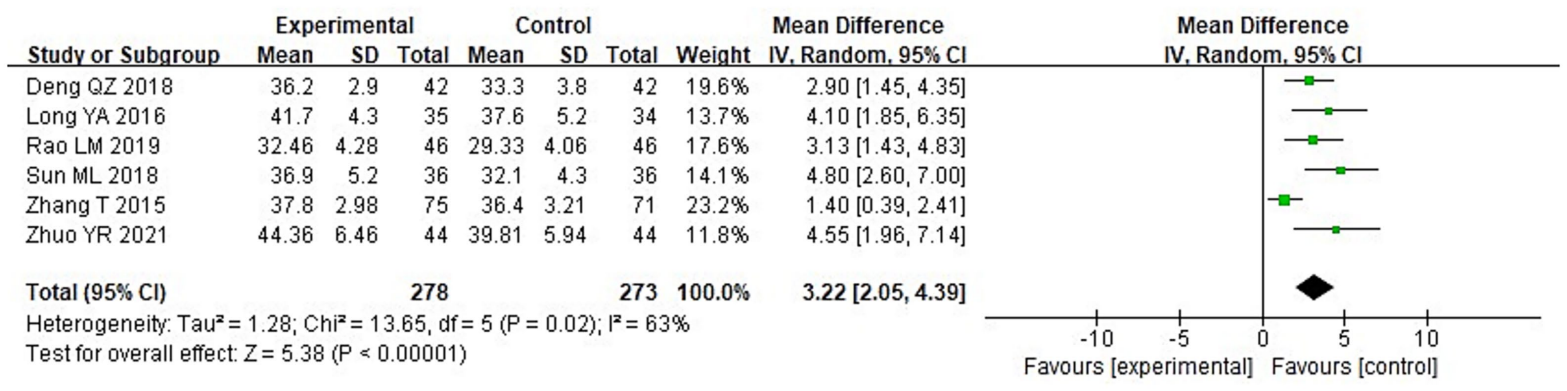
Forest analysis diagram comparing the levels of albumin (Alb) in the two groupings.

**Figure 8:**
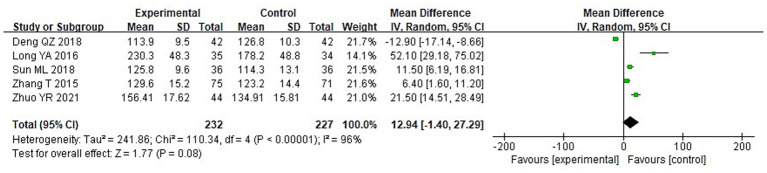
Forest analysis diagram comparing the levels of hemoglobin (Hb) in the two groupings.

**Figure 9:**
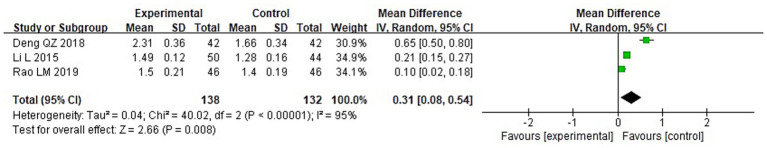
Forest analysis diagram comparing the total blood lymphocyte count (TLC) of the two groupings.

A meta-analysis was carried out to assess the post-treatment nutritional status indicators in both groupings. The heterogeneity test findings were as follows: PA: Chi^2^ = 47.88, df = 2, *p* < 0.00001, I^2^ = 96% (Figure 6); Alb: Chi^2^ = 13.65, df = 5, *p* = 0.02, I^2^ = 63%; Hb: Chi^2^ = 110.34, df = 4, *p* < 0.00001, I^2^ = 96%; and TLC: Chi^2^ = 40.02, df = 2, *p* < 0.00001, I^2^ = 95%. These results indicate substantial heterogeneity among the included studies. Using a random-effects model, the analysis demonstrated that serum levels of Hb and TLC were considerably higher in the IG in contrast to the CG (*p* < 0.05) (Figures 7–9). Although serum PA levels were also elevated in the IG, the disparity wasn’t that great (*p* > 0.05).

#### 3.3.4 Immune function

**Figure 10:**
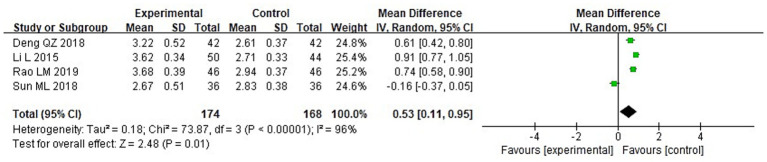
Forest analysis diagrams comparing the levels of IgA in the two groupings.

**Figure 11:**
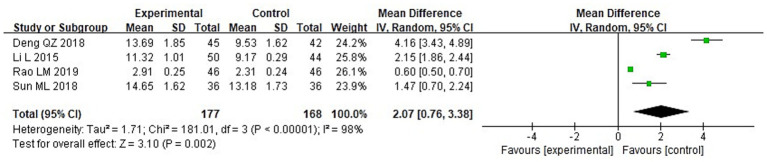
Forest analysis diagram comparing the IgM levels of the two groupings.

**Figure 12:**
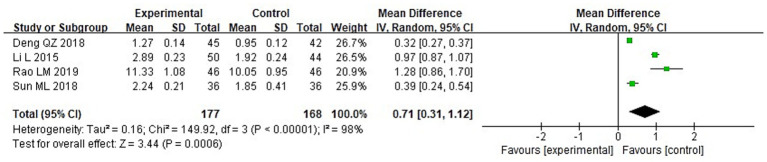
Forest analysis plot comparing the IgG levels of the two groups.

A meta-analysis was conducted to evaluate immune function indicators in both groupings following treatment. The results of the heterogeneity tests were as follows: IgA: Chi^2^ = 73.87, df = 3, *p* < 0.00001, I^2^ = 96%; IgM: Chi^2^ = 181.01, df = 3, *p* < 0.00001, I^2^ = 98%; and IgG: Chi^2^ = 149.92, df = 3, *p* < 0.00001, I^2^ = 98%. These findings indicate substantial heterogeneity across the included studies. Using a random-effects model, the analysis demonstrated that post-treatment serum levels of IgA, IgG, and IgM were considerably higher in the IG in contrast to the CG (*p* < 0.05) (Figures 10–12).

#### 3.3.5 Serum inflammatory factors

**Figure 13:**
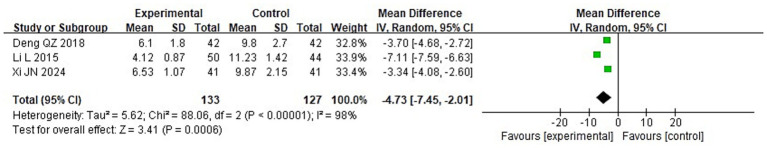
Forest analysis chart comparing the levels of serum IL-2 in the two groupings.

**Figure 14:**
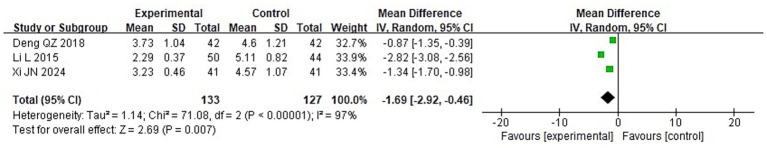
Forest analysis chart comparing the levels of serum IL-6 in the two groupings.

**Figure 15:**
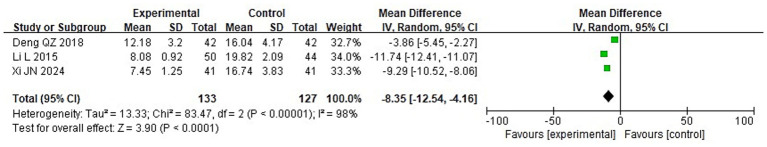
Forest analysis chart comparing the levels of serum TNF-*α* in the two groups.

To compare the two groups’ post-treatment levels of inflammatory cytokines, a meta-analysis was carried out. The following were the findings of the heterogeneity test: Chi^2^ = 88.06, df = 2, *p* < 0.00001, I^2^ = 98% for IL-2; Chi^2^ = 71.08, df = 2, *p* < 0.00001, I^2^ = 97% for IL-6; and Chi^2^ = 84.37, df = 2, *p* < 0.00001, I^2^ = 98% for TNF-*α*. These findings suggest that the included studies exhibit significant heterogeneity. Following treatment, the IG’s blood levels of IL-2, IL-6, and TNF-α were considerably lower than those of the CG (*p* < 0.05), according to analysis using a random-effects model (Figures 13–15).

#### 3.3.6 Exploration of heterogeneity (subgroup and sensitivity analyses)

Several pooled outcomes (nutritional status markers, immunoglobulins, and inflammatory cytokines) exhibited very high heterogeneity (I^2^ > 95%). To explore potential sources, we conducted prespecified sensitivity analyses: Leave-one-out analyses did not identify a single outlier study driving the heterogeneity; pooled effects remained directionally consistent. Excluding studies with distinct control regimens (e.g., trials that used family nutrition management as control rather than standard hospital-based care) qualitatively reduced between-study variability for immune and inflammatory markers, although substantial heterogeneity persisted. Restricting to similar intervention routes (EN-only vs. nasogastric nutrition management) attenuated dispersion in several biomarker outcomes but did not fully resolve inconsistency. Excluding the hemorrhagic-stroke-only cohort with dysphagia yielded more homogeneous estimates for some inflammatory markers, suggesting stroke subtype may contribute to heterogeneity.

#### 3.3.7 Incidence of infectious complications

**Figure 16:**
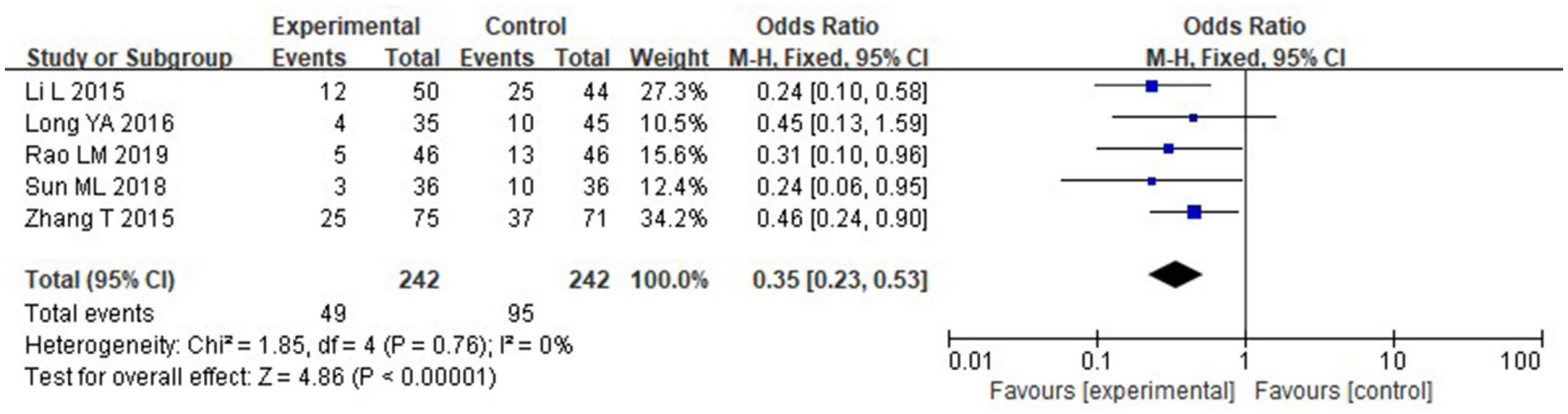
Forest analysis chart comparing the incidence of infection complications in the two groups.

Five of the studies that were considered provided information on the prevalence of infectious complications. The following outcomes were obtained using the heterogeneity test: With chi^2^ = 1.85, df = 4, *p* = 0.76, and I^2^ = 0%, there is no discernible variation among the studies. The fixed-effect model was thus used. According to the meta-analysis, the IG experienced a considerably reduced incidence of infectious complications than the CG (*p* < 0.05) (Figure 16).

Consistency of definitions and assessment methods for infectious complications, among the five included trials reporting infectious complications, the definitions and diagnostic criteria were not fully standardized. Three studies defined infections based on clinical and laboratory evidence of pneumonia, urinary tract infection, or sepsis, following hospital-based diagnostic criteria (12–14). Two studies (15, 16) did not specify diagnostic criteria in detail but described infection as “fever with elevated inflammatory markers and confirmed bacterial infection.” None of the studies clearly indicated whether microbiological confirmation or radiographic evidence was routinely required.

The assessment timeframes also varied, ranging from hospitalization period only (12, 13) to up to 4 weeks post-treatment (17). Moreover, differences in infection classification (e.g., respiratory vs. systemic infections) were not consistently reported. This inconsistency in definitions and detection methods likely influenced the pooled estimate of infection incidence, despite the statistical homogeneity observed (I^2^ = 0%).

To improve transparency, a summary of infection assessment definitions across included studies is provided in Table 2.

**Table 2:** Variability in definitions and methods used for assessing infectious complications across included studies.

Study (first author, year)	Infection definition	Diagnostic basis	Assessment period	Infection types reported
Li Li, 2015	Nosocomial infections during hospitalization	Clinical + lab (WBC↑, CRP↑, fever)	During hospital stay	Pneumonia, urinary tract infection
Rao Liumei, 2019	Early postoperative infections	Clinical + lab + physician diagnosis	≤14 days	Pneumonia
Zheng T., 2015	Pulmonary or systemic infection	Clinical symptoms + lab + imaging	≤4 weeks	Pneumonia, sepsis
Sun Maling, 2018	“Fever with elevated inflammatory markers”	Clinical only	Unclear	Not specified
Long Youai, 2016	Infection complications	Clinical only	≤2 weeks	Not specified

### 3.4 Subgroup and sensitivity analyses

To explore potential sources of the considerable heterogeneity (I^2^ > 95%) observed across several pooled outcomes, subgroup and sensitivity analyses were performed. (1) By nutritional support type: When studies were stratified by intervention type, the EN-only subgroup demonstrated consistent improvements in nutritional (Alb, PA) and immune markers (IgG, IgA) with reduced heterogeneity (I^2^ ranging from 60 to 75%). In contrast, trials combining enteral and parenteral nutrition (EN + PN) or including immunonutrient supplementation retained high heterogeneity (I^2^ > 90%), suggesting compositional variability contributed to between-study differences. (2) By stroke type: Subgrouping by stroke subtype (ischemic vs. hemorrhagic) revealed that studies enrolling only hemorrhagic stroke patients (17) showed stronger effects on serum inflammatory cytokines (TNF-*α*, IL-6 reduction), whereas mixed or ischemic cohorts exhibited moderate effects with persistent heterogeneity (I^2^ > 85%). (3) By timing of nutritional support: Early nutritional intervention (initiated within 72 h post-stroke) yielded greater improvements in GCS, PA, and IgG levels compared with delayed initiation (>72 h), and heterogeneity was partially attenuated (I^2^ = 70–80%). These results suggest that intervention timing may explain some between-study variation. (4) Sensitivity analyses: Leave-one-out analyses indicated that no single study disproportionately affected pooled effect sizes. After excluding studies rated as high risk for performance or detection bias, the overall direction and significance of results remained unchanged, though heterogeneity decreased modestly (by 10–15%) for most biochemical outcomes.

Despite these subgroup efforts, residual heterogeneity remained substantial (I^2^ > 80%) in some outcomes (e.g., IL-2, TNF-*α*), implying unmeasured methodological and clinical variability. Detailed subgroup results and corresponding forest plots are presented in Supplementary Figures S1–S3.

## 4 Discussion

Stroke is a neurological disorder resulting from cerebrovascular injury and subsequent brain tissue necrosis, characterized by high incidence and mortality (18). In severe cases, rapid disease progression, impaired consciousness, motor dysfunction, and gastrointestinal dysmotility often lead to dysphagia and negative nitrogen balance (19, 20). The present meta-analysis synthesized data from eight randomized controlled trials evaluating nutritional support combined with conventional therapy in stroke patients. Consistent with prior reports, nutritional intervention significantly improved short-term nutritional and immune indicators (PA, Hb, TLC, IgA, IgG, IgM) and reduced inflammatory cytokines (IL-2, IL-6, TNF-*α*), thereby lowering infection risk. However, its effect on neurological function as measured by NIHSS was limited, suggesting that the primary benefits of nutritional support occur during early metabolic stabilization rather than direct neurofunctional recovery.

Based on the findings of this meta-analysis, nutritional support demonstrated significant benefits across multiple clinical domains in stroke patients, though with notable variations in treatment effects. The intervention group showed consistent improvements in nutritional biomarkers including hemoglobin, total lymphocyte count, and immunoglobulins, with elevated prealbumin levels indicating effective protein-energy supplementation. These nutritional improvements were accompanied by enhanced immune competence, as evidenced by increased cellular and humoral immunity markers, which likely contributed to the observed reduction in infectious complications. Simultaneously, significant reductions in pro-inflammatory cytokines (IL-2, IL-6, TNF-*α*) were observed, suggesting effective modulation of the systemic inflammatory response, possibly mediated through immunonutrients such as *ω*-3 fatty acids and arginine used in some trials. However, neurological outcomes revealed a divergent pattern: while Glasgow Coma Scale scores improved significantly, indicating better consciousness levels potentially related to metabolic stabilization, NIH Stroke Scale scores showed no statistically significant improvement, suggesting that nutritional support may primarily affect arousal and alertness rather than higher-order neurological functions within short-term follow-up periods. Substantial heterogeneity (I^2^ > 90%) was noted across several outcomes, potentially arising from variations in nutritional support routes, formula compositions, initiation timing, patient characteristics, and control interventions, with studies focusing on hemorrhagic stroke patients demonstrating more pronounced anti-inflammatory effects compared to mixed cohorts.

These results align with previous meta-analyses and clinical studies. Fu et al. (9) and Khoshbonyani et al. (5) also reported that nutritional therapy reduces post-stroke infections and improves metabolic homeostasis, while its influence on long-term neurological outcomes remains modest. Ikezawa et al. (6) similarly observed that early enteral nutrition enhances discharge readiness through improved immune and metabolic status rather than direct neurological repair. Collectively, these findings emphasize that nutritional interventions primarily stabilize systemic metabolism, attenuate inflammation, and prevent complications during the acute phase of stroke.

Nutritional adequacy also plays an essential role in maintaining immune competence and physical performance in healthy populations (21), further underscoring its physiological importance for recovery in stroke patients.

The present analysis identified significant improvement in GCS but only borderline NIHSS changes. This discrepancy reflects the differing physiological domains of the two scales: the GCS assesses consciousness and arousal, which respond to early metabolic stabilization, whereas the NIHSS measures higher-order cortical functions requiring longer neuroplastic recovery. Mechanistically, early nutritional support may improve consciousness via enhanced glucose and lipid metabolism, reduced oxidative stress, and preservation of the blood–brain barrier. In contrast, improvements in motor and language function depend on longer-term neuronal remodeling. Thus, GCS may serve as a sensitive short-term endpoint, while NIHSS remains the benchmark for long-term neurological recovery.

Increases in PA, Hb, and TLC levels confirmed that nutritional support can quickly improve protein–energy malnutrition and immune competence. PA, with its short half-life, sensitively reflects early nutritional improvement; higher Hb suggests correction of inflammatory anemia, and increased TLC reflects restored cellular immunity, contributing to lower infection rates. Enhanced immunoglobulin levels (IgA, IgG, IgM) further indicate strengthened humoral immunity. Meanwhile, the observed reductions in TNF-*α*, IL-2, and IL-6 suggest that early nutritional therapy attenuates systemic inflammation, thereby preventing further ischemic damage and secondary infection.

Although subgroup analyses by nutrition type, stroke subtype, and timing partially reduced variability, substantial heterogeneity persisted (I^2^ > 80%). This heterogeneity likely reflects clinical and methodological differences, including formula composition, caloric targets, intervention duration, and patient comorbidities. Variability in measurement protocols and incomplete descriptions of enteral/parenteral ratios and nutrient formulations further limited comparability. Even after sensitivity analyses excluding high-risk studies, heterogeneity remained high, indicating genuine diversity among study designs. Future RCTs should adopt standardized definitions of nutritional interventions and outcome measures to enable more robust quantitative synthesis. The heterogeneity likely arises from: (1) differences in intervention route and composition (EN vs. nasogastric; potential use of immunonutrients), (2) timing and duration of nutritional support (early/acute-phase initiation vs. later; short vs. longer courses), (3) patient mix (hemorrhagic vs. ischemic/mixed; baseline severity and nutritional risk), (4) control care pathways (“conventional care” vs. “family nutrition management”), and (5) assay protocols for laboratory outcomes.

The current review has several limitations that warrant consideration. First, all included RCTs exhibited methodological weaknesses, particularly the absence of blinding and incomplete reporting of follow-up data, which introduces risks of performance and detection bias. Second, substantial heterogeneity was observed across multiple outcomes, which could be attributed to variations in nutritional protocols, patient characteristics, and measurement methods—though prespecified subgroup and sensitivity analyses were performed to explore these sources. Furthermore, inconsistencies in the definitions and assessment methods for infectious complications across the included studies may influence the validity of the pooled estimate for infection incidence, despite the statistical homogeneity observed. Although the beneficial direction of nutritional support was consistently demonstrated, the magnitude of effect may be overestimated. In addition, all studies focused on short-term outcomes (generally 2–4 weeks post-intervention) and lacked long-term follow-up data on critical endpoints such as mortality, functional independence, or quality of life. Finally, the small number of included studies precludes a reliable assessment of publication bias. Consequently, the overall certainty of evidence is judged to be low to very low.

Despite these limitations, the consistent improvement across nutritional, immune, and inflammatory markers indicates a physiologically meaningful benefit during the acute phase of stroke. Future multicenter, blinded RCTs with standardized protocols and long-term follow-up are warranted to confirm these findings and clarify their clinical implications.

## 5 Conclusion

In summary, the current evidence suggests that nutritional support therapy, as an adjunct to conventional treatment, may confer benefits in improving short-term nutritional status, enhancing immune function, and reducing infectious complications in stroke patients. However, its effects on neuromotor recovery remain inconclusive. These findings must be interpreted with caution due to substantial methodological limitations observed across the included studies, including the absence of blinding, incomplete follow-up reporting, and significant variability in nutritional intervention protocols. The overall certainty of evidence is rated as low to very low. Future rigorously designed randomized controlled trials featuring standardized nutritional regimens, adequate blinding procedures, and longer-term outcome assessments are warranted to validate these preliminary findings and establish evidence-based clinical guidelines.

## Supplementary material

Supplementary Figure S1 Subgroup analysis by nutritional support type (EN vs. EN+PN) for nutritional and immune outcomes.

Supplementary Figure S2 Subgroup analysis by stroke subtype (hemorrhagic vs. ischemic/mixed) for inflammatory markers.

Supplementary Figure S3 Sensitivity analyses excluding high-risk-of-bias studies for robustness evaluation.
