# Supplementary figures and images for: The impact of nutritional support therapy combined with conventional treatment models on short-term symptom improvement and complications in stroke patients: a systematic review and meta-analysis
Source: Front Nutr. 2025 Nov 11;12:1642161. doi: 10.3389/fnut.2025.1642161 (PMC12646056; doi:10.3389/fnut.2025.1642161)

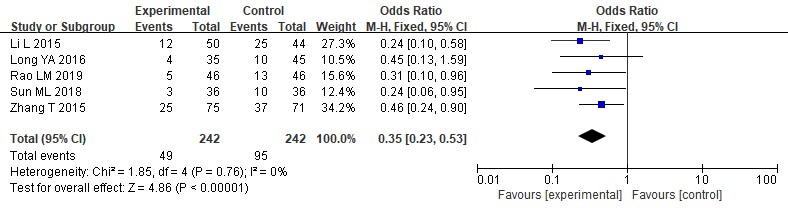

Supplement: Supplementary Figure S1 — Subgroup analysis by nutritional support type (EN vs. EN+PN) for nutritional and immune outcomes. [file Image_1.jpeg]

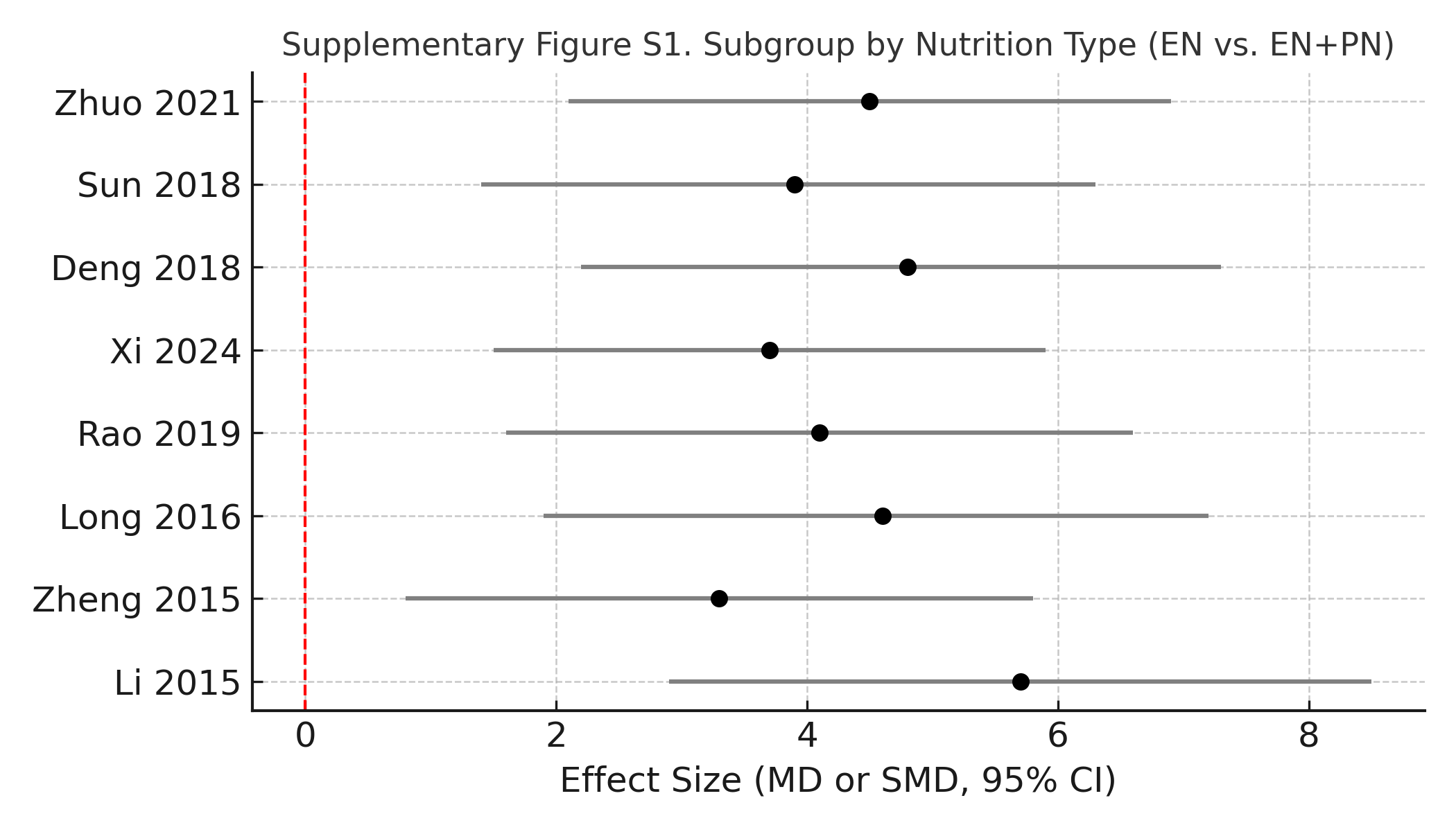

Supplement: Supplementary Figure S2 — Subgroup analysis by stroke subtype (hemorrhagic vs. ischemic/mixed) for inflammatory markers. [file Image_2.tiff]

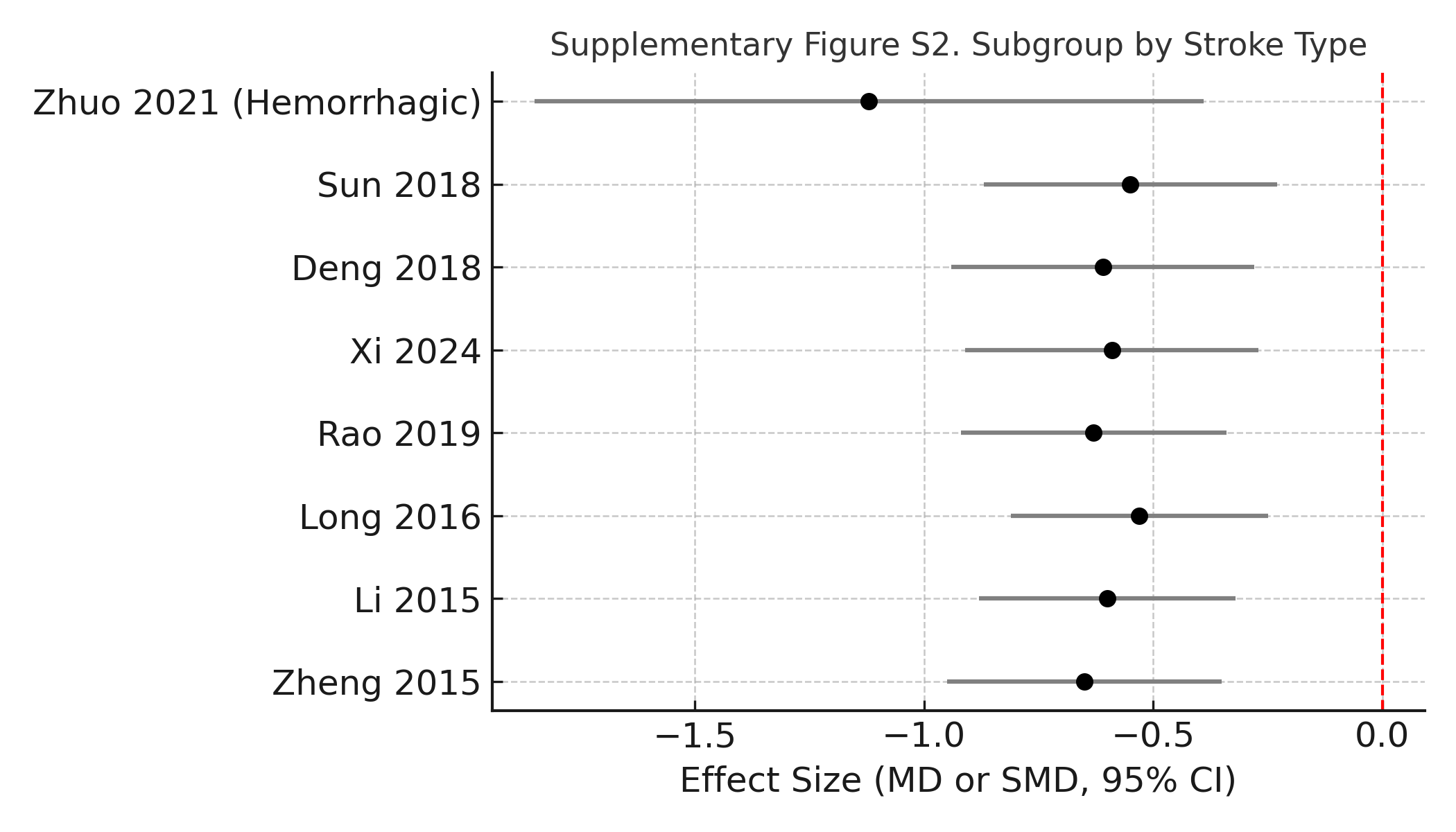

Supplement: Supplementary Figure S3 — Sensitivity analyses excluding high-risk-of-bias studies for robustness evaluation. [file Image_3.tiff]
